# Ficin-Loaded Quaternized Chitosan Hydrogels: A Functional Biobased Platform for Enhanced Hemostasis

**DOI:** 10.3390/polym18212679

**Published:** 2026-10-28

**Authors:** Maria S. Lavlinskaya, Andrey V. Sorokin, Yulia A. Redko, Victoria A. Koroleva, Maxim S. Kondratyev, Yuriy F. Zuev, Marina G. Holyavka, Valeriy G. Artyukhov

**Affiliations:** 1 Biophysics and Biotechnology Department, Voronezh State University, 1 Universitetskaya Square, Voronezh 394018, Russia; maria.lavlinskaya@gmail.com (M.S.L.); andrew.v.sorokin@gmail.com (A.V.S.); redkoju@yandex.ru (Y.A.R.); koroleva_victoria@bk.ru (V.A.K.); ma-ko@bk.ru (M.S.K.); artyukhov@bio.vsu.ru (V.G.A.); 2 Institute for Advanced Studies, Sevastopol State University, 33 Universitetskaya Street, Sevastopol 299053, Russia; 3 Biology Department, Nikolay Nikolaevich Burdenko Voronezh State Medical University, 10 Studencheskaya Street, Voronezh 394036, Russia; 4 Laboratory of Structure and Dynamics of Biomolecular Systems, Institute of Cell Biophysics of the Russian Academy of Sciences, 3 Institutskaya Street, Pushchino 142290, Russia; 5 Kazan Institute of Biochemistry and Biophysics, Federal Research Center “Kazan Scientific Center of the Russian Academy of Sciences”, 2/31 Lobachevsky Street, Kazan 420111, Russia; yufzuev@mail.ru

**Keywords:** *N*-(2-Hydroxypropyl)-3-trimethylammonium chitosan, hydrogel, ficin, immobilization, hemostasis, blood coagulation, protein–polysaccharide complexes

## Abstract

Biopolymer-based hydrogels are promising platforms for hemostatic applications, yet most chitosan-based materials rely on passive plug formation rather than activation of the natural coagulation cascade. This study aimed to develop hemostatic hydrogels based on quaternized chitosan that activate the coagulation cascade through immobilized ficin. *N*-(2-Hydroxypropyl)-3-trimethylammonium chitosan (HTCC) with varying degrees of quaternization was synthesized and crosslinked with 1,4-butanediol diglycidyl ether to obtain Cross-HTCC hydrogels. The hydrogels were characterized by FTIR spectroscopy, ζ-potential measurements, and swelling tests. Ficin was immobilized by adsorption, and the effects of pH, contact time, and protein content on immobilization efficiency and proteolytic activity were examined. Ficin release and whole-blood coagulation were also assessed. The hydrogels exhibited ζ-potential values above 30 mV and swelling ratios of up to 28.9-fold. Ficin immobilization was optimal at pH 7.4, with maximum sorption reached after ~180 min. Ficin interacted with heterogeneous sorption sites through hydrogen bonding, electrostatic, and hydrophobic interactions, as confirmed by isotherm fitting, FTIR analysis, and molecular dynamics simulations. The immobilized enzyme was released within 24 h and significantly accelerated blood clotting compared with ficin-free hydrogels. These findings demonstrate that ficin-loaded Cross-HTCC hydrogels combine efficient enzyme loading with enhanced hemostatic performance and hold promise as multifunctional hemostatic materials.

## 1. Introduction

Biopolymer-based hydrogels have emerged as versatile platforms for a wide range of bio-related applications, including drug delivery [1], wound healing [2,3], antibacterial and antifungal therapy [4,5], antiviral treatment [6], hemostasis [7], tissue engineering [8], and bone regeneration [9]. Their appeal lies in a combination of availability, low toxicity, biocompatibility, biodegradability, and intrinsic antibacterial activity.

Among biopolymers, chitosan—a polyaminosaccharide obtained by deacetylation of chitin—stands out as one of the most widely used biomaterials in biomedicine [10]. However, despite its versatility, chitosan has several drawbacks that limit its biomedical use. The first is limited solubility: native chitosan is practically insoluble in aqueous media at pH > 6.5 because its amino groups are deprotonated, which reduces its hydrophilicity. In addition, chitosan consists of many polymer chains held together by a network of intermolecular hydrogen bonds, which hinder the solvent interaction required for dissolution. The second drawback is poor tissue adhesion. Chitosan contains a large number of OH and NH_2_ groups, but strong bioadhesion requires other functional groups that can either form covalent bonds with tissue proteins or enhance electrostatic interactions with the negatively charged surfaces of tissues and cells.

Both drawbacks can be addressed through chemical modification by introducing quaternary ammonium groups into chitosan macromolecules. Such modification is particularly attractive for biomedical applications, as it improves not only solubility and tissue adhesion but also antibacterial activity [11]. For example, Dhlamini et al. showed that *N*-(2-hydroxy)propyl-3-trimethylammonium chitosan chloride (HTCC) powder exhibits greater antibacterial activity against *S. aureus* than native chitosan, and HTCC nanoparticles show even higher activity than the powder [12]. This finding indicates that the form in which chitosan or its modified derivatives are applied is also critical for achieving the desired biomedical effect.

In this context, hydrogels—with their hydrophilic, elastic, highly swelling, and porous structure—are indispensable when a material must absorb significant amounts of fluid, maintain moisture levels, and support cell proliferation [13]. These properties are particularly valuable for developing multifunctional hemostatic agents. Hydrogels based on quaternized chitosan are especially promising in this regard.

Several recent studies illustrate this potential. Wei et al. developed HTCC–dialdehyde cellulose composites that combine reported antibacterial activity with strong blood coagulation ability, reducing clotting time by 76.6% and 59.8% relative to the blank control and Celox™, respectively, while maintaining low hemolysis rates [14]. A sponge based on quaternized chitosan and carboxylated cellulose nanofibers reduced bleeding time by more than 2.5-fold in a mouse tail vein injury model and was reported to accelerate wound healing through its antibacterial effect [15]. An HTCC-based hydrogel containing zinc ions reduced blood clotting time by approximately 16-fold compared with whole blood, with antibacterial activity attributed to both the polycationic nature of HTCC and the release of zinc ions [16].

These and many other studies reflect the general trends in modern hemostatic hydrogel development based on functionalized chitosan. Most work focuses on enhancing the antibacterial effect of chitosan (e.g., by incorporating zinc ions [16] or silver nanoparticles [17]) or on exploiting the positive charge of quaternized chitosan to accelerate blood cell aggregation and initial thrombus formation.

However, relatively few studies attempt to accelerate hemostasis not only through the external charge or structural properties of quaternized chitosan hydrogels (a plug that compresses the damaged vessels) but also by activating the natural hemostatic cascade. One example is the immobilization of blood coagulation factors on chitosan hydrogels [18]. This limited number of studies is largely due to the high cost of protein coagulation factors such as thrombin and fibrinogen, which would make the resulting product poorly accessible to consumers. There is therefore a need for a more accessible component that can activate the hemostatic cascade, bind to a chitosan hydrogel, and be released upon contact with the wound.

Ficin (EC 3.4.22.3), a plant cysteine protease, is a promising candidate. Owing to its high proteolytic activity and broad substrate specificity [19], ficin has been reported to activate human coagulation factor X [20], which may enable the creation of multifunctional quaternized chitosan hemostatic agents that activate the natural hemostatic cascade. Ficin has also been described as possessing antibacterial and antibiofilm properties, which further expands the functionality of the resulting hemostatic agent [21]. However, ficin is sensitive to its microenvironment, and its activity depends on both the pH of the medium and its content within the hemostatic agent. As a protease, ficin is prone to autolysis, the rate of which increases with ficin concentration [22]. Careful optimization of the conditions for incorporating ficin into a quaternized chitosan hydrogel is therefore required.

To address the existing gap in the development of multifunctional chitosan hemostatic agents that initiate the natural hemostatic cascade through coagulation factor activators, the present work aimed to develop hemostatic hydrogels based on quaternized chitosan that activate the natural coagulation cascade through immobilized ficin. To this end, HTCC hydrogels with varying degrees of quaternization and crosslinking were synthesized, the conditions for ficin immobilization were optimized, the interaction of ficin with the hydrogels was studied, and the potential of the resulting compositions was demonstrated in whole-blood kinetic coagulation experiments.

## 2. Materials and Methods

### 2.1. Materials

Chitosan (Cs; molecular weight 200 kDa, degree of deacetylation 0.91; Bioprogress, Moscow, Russia), glycidyltrimethylammonium chloride (GTMAC; >95%), and 1,4-butanediol diglycidyl ether (>98%; both from Shanghai Macklin Biochemical Technology Co., Ltd., Shanghai, China) were used to synthesize HTCCs and HTCC-based hydrogels. Ficin (F4165), bovine serum albumin (BSA; A9647), and azocasein (A2765) were purchased from Sigma-Aldrich (St. Louis, MO, USA). Acetone, methanol, and ethanol (all chemical grade) were provided by Vekton-Center (St. Petersburg, Russia). All reagents were used as received.

### 2.2. Synthesis of N-(2-Hydroxypropyl)-3-trimethylammonium Chitosan Chloride (HTCC) with Varying Degrees of Quaternization

Chitosan (Cs; 3 g, 18.5 mmol) was placed in a thermostated reactor equipped with an overhead stirrer and a reflux condenser and dispersed in 30 mL of water at 85 °C. The calculated amount of glycidyltrimethylammonium chloride (GTMAC; Table 1) was then added in three equal portions at 1 h intervals. Two hours after the final addition, the solution was precipitated into cold acetone (500 mL) and left overnight at 4 °C. Acetone was decanted, and the resulting precipitate was dissolved in methanol (100 mL). The solution was reprecipitated into an acetone–ethanol mixture (4:1 *v*/*v*, 300 mL). The precipitate was collected using a Büchner funnel and dried in a vacuum oven at 45 °C to constant weight.

### 2.3. Synthesis of HTCC-Based (Cross-HTCC) Hydrogels

HTCC (1 g) and 40 mL of water were placed in a thermostated reactor equipped with a stirrer and a reflux condenser. After the chitosan had completely dissolved, 0.875 or 1.75 mL of 1,4-butanediol diglycidyl ether (BDDE) was added to obtain Cross-HTCC-1-1 (*n* (HTCC):*n* (BDDE) = 1:1) and Cross-HTCC-1-2 (*n* (HTCC):*n* (BDDE) = 1:2), respectively. *n* (HTCC) refers to the number of moles of monomeric sugar units. The mixture was kept at 80 °C for 2 h. Once the reaction was complete, the resulting gel was collected and purified by dialysis against distilled water through a regenerated cellulose membrane (molecular weight cut-off 3.5 kDa) for three days. The product was then freeze-dried to constant weight, with a yield of 85–89%.

### 2.4. Instrumental Characterization of HTCCs and Cross-HTCC Hydrogels

Fourier-transform infrared (FTIR) spectroscopy was used to confirm the chitosan modification. FTIR spectra of dry powders, pre-ground in a vibration mill, were recorded in the 4000–900 cm^−1^ range on a Shimadzu IRAffinity-1 FTIR spectrophotometer (Shimadzu Scientific Instruments, Kyoto, Japan) in attenuated total reflectance mode.

The degree of quaternization of the HTCCs was determined by ^1^H NMR spectroscopy using pulsed field gradient diffusion filtering. Spectra were recorded at 30 °C on an AVANCE 600 instrument (Bruker Corporation, Billerica, MA, USA). The concentration of HTCCs was 10 mg/mL, and the solvent was 2% (*v*/*v*) CD_3_COOD + 98% (*v*/*v*) D_2_O. Further details are provided in the Supplementary Materials.

The ζ-potential of the HTCCs and Cross-HTCCs was measured on 0.05% (*w*/*v*) aqueous solutions using a Zetasizer Nano ZS instrument (Malvern Panalytical B.V., Almelo, The Netherlands).

### 2.5. Evaluation of the Swelling Ratio of Cross-HTCC Hydrogels

The swelling behavior of the Cross-HTCCs was studied in distilled water and in a BSA solution (6 g/dL in 0.15 M NaCl) that mimics the blood environment. A Cross-HTCC sample (~0.01 g) was placed in 10 mL of the respective medium and kept at 25 °C for 24 h. The polymer was then filtered off, the excess liquid was removed from its surface, and the sample was weighed. The swelling ratio (SR) was calculated as [23]:

(1) $$\mathrm{SR}=\frac{m_{1}-\;m_{0}}{m_{0}},$$

where *m*_1_ and *m*_0_ are the masses of the swollen and dry hydrogel, respectively (g).

### 2.6. Ficin Immobilization on Cross-HTCC Hydrogels

Ficin was immobilized by adsorption as follows. A Cross-HTCC hydrogel (20 mg) was incubated in 4 mL of ficin solution (0.05 M Tris-HCl buffer, pH 7.4, or 0.05 M borate buffer with 0.1 M KCl, pH 9.0) at concentrations, *C_sol_*, ranging from 0.0725 mg/mL (3 µM) to 3.63 mg/mL (152 µM) for a defined period at 25 °C. The sorbent was then separated from the enzyme solution and washed with buffer in 20 mL portions until no protein signal was detected at λ = 280 nm using an SF-2000 spectrophotometer (LOMO Systems, St. Petersburg, Russia). The protein content in the immobilized preparation, *C_ads_*, was calculated as:

(2) $$C_{\mathrm{ads}}=\frac{{(C}_{0}-\;C_{1})V}{m},$$

where *C*_0_ and *C*_1_ are the ficin concentrations before and after sorption, respectively (mg/mL), *V* is the volume of the ficin solution (mL), and *m* is the mass of Cross-HTCC hydrogel (g).

Protein concentrations in solutions were measured by the Lowry protein assay [24]. Throughout the text, the data are expressed as the amount of adsorbed protein per gram of Cross-HTCC hydrogel.

Total protease activity in the azocasein hydrolysis reaction was assessed as described in [25].

### 2.7. Ficin Adsorption Isotherms

To investigate the adsorption behavior of ficin on the hydrogel surface, the dependence of ficin content in the hydrogels on the concentration of the feed solution was fitted with the Langmuir and Freundlich isotherm models. The Langmuir equation in the Hanes–Woolf linearization is described by [26]:

(3) $$\frac{C_{\mathrm{sol}}}{C_{\mathrm{ads}}}=\;\frac{1}{Q_{\max}K_{L}}+\frac{C_{\mathrm{sol}}}{Q_{\max}},$$

where *Q_max_* is the maximum monolayer capacity (mol/g), and *K_L_* (L/mol) is the Langmuir equilibrium constant.

The Freundlich isotherm in linear form is given by [27]:

(4) $$\ln C_{\mathrm{ads}}=\ln K_{F}+\frac{C_{\mathrm{sol}}}{n},$$

where *K_F_* is the adsorption capacity constant (L/mol), and *n* is an empirical parameter reflecting the degree of surface heterogeneity.

### 2.8. Ficin Adsorption and Release Kinetics

To identify the rate-limiting step of ficin sorption, the kinetic results were fitted with mathematical models. The pseudo-first-order kinetic model describes a linear relationship between the reaction rate and the sorbate concentration. In this model, sorption is controlled by diffusion of the sorbate across the sorbent–solution interface and is described by [28]:

*ln*(*Q_e_* − *Q_t_*) = *lnQ_e_* − *k*_1*s*_*t*, (5)

The pseudo-second-order kinetic model assumes that chemisorption is the rate-limiting step and that the amount of sorbed species correlates with the number of available active sites on the sorbent surface [29]:

(6) $$\frac{t}{Q_{t}}=\;\frac{1}{k_{2s}Q_{e}^{2}}+\;\frac{t}{Q_{e}},$$

The Ritger–Peppas model is used to determine the type of sorbate diffusion within the sorbent phase. Notably, to obtain more reliable results, this model should be applied only to data from the early stages of the process, i.e., when the amount of sorbed species does not exceed 60% of its maximum value. The Ritger–Peppas model is expressed as [30]:

(7) $$\frac{Q_{t}}{Q_{e}}=k\times t^{n_{s}},$$

where *Q_e_* (mg/g) and *Q_t_* (mg/g) represent the amount of adsorbed ficin at equilibrium and at time *t*, respectively; *k*_1*s*_ and *k*_2*s*_ (min^−1^) are the rate constants for the pseudo-first-order and pseudo-second-order models, respectively; *k* is the structural parameter; and *n_s_* is the exponent indicating the type of diffusion mechanism.

To gain deeper insight into the release behavior of ficin from the hydrogel compositions, the experimental data were fitted with first-order release kinetics and the Korsmeyer–Peppas model.

The first-order release kinetic model assumes that changes in the concentration of the released species over time depend only on its initial value. The model is described by [31]:

(8) $$\log q_{t}=\log q_{0}+\;\frac{k_{1r}t}{2.303},$$

where *q*_0_ (mg) and *q_t_* (mg) are the amounts of substance in the polymer and the amount released at time *t*, respectively, and *k*_1*r*_ (min^−1^) is the first-order release rate constant.

The Korsmeyer–Peppas model is used to assess the type of transport of the solvent or the released species within the carrier matrix, as well as the contribution of diffusion to the overall release process. It is described by [32]:

(9) $$\frac{q_{t}}{q_{\infty }}=k_{\mathrm{KP}}t^{n_{r}},$$

where *qt* (mg) and *q*∞ (mg) are the amounts of released substance at time *t* and at equilibrium, respectively; *k_KP_* is a structural parameter; and *n_r_* is the power-law exponent reflecting the type of transport and diffusion of the released species.

### 2.9. Molecular Dynamics (MD) Simulation

The three-dimensional structure of ficin (PDB ID: 8R78) was retrieved from the RCSB Protein Data Bank (https://www.rcsb.org, accessed on 24 September 2026). To prepare each hydrogel molecule for calculations, its structure was drawn and initially optimized using the HyperChem 8.0 chemical editor (http://www.hypercubeusa.com/, accessed on 24 September 2026). This step was performed independently by two research groups to minimize the likelihood of errors in the molecular structures. The resulting models were then subjected to the AMBER10 force field within the NAMD 2.14 software package (https://www.ks.uiuc.edu/Research/namd/, accessed on 24 September 2026), where they underwent further geometric and energetic optimization. The enlarged carrier grids contained 3542 atoms for Cross-HTCC-4-1-1 and 4402 atoms for Cross-HTCC-3-1-1. In NAMD, while preparing each complex for molecular dynamics calculations, the cross-HTCC molecules were placed at the center of a cube, and the remaining space was filled with approximately 50,000 H_2_O molecules along with Na^+^ and Cl^−^ ions. The entire system, comprising approximately 60,000 atoms, was then equilibrated for 100 ps. The subsequent primary MD simulation yielded a 10 ns molecular dynamics trajectory at a pressure of 1 atm and a temperature of 300 K, using the AMBER10:EHT force field in NAMD 2.14. System behavior was analyzed using the VMD 2.0 software package (https://www.ks.uiuc.edu/Research/vmd/, accessed on 24 September 2026).

### 2.10. Blood Sampling

Venous blood was obtained from healthy volunteers aged 18–45 years. All procedures complied with good clinical practice and the ethical requirements for blood collection. The study followed the principles of the Declaration of Helsinki (7th revision, 2013) and the ethical guidelines of the Voronezh Regional Blood Center. Blood was pretreated with an anticoagulant mixture (citric acid, sodium citrate, and dextrose).

### 2.11. Kinetic Whole Blood Coagulation

Kinetic whole-blood coagulation was performed at 25 °C according to the procedure described in [33]. Briefly, samples of ficin, Cross-HTCC hydrogels or Ficin + Cross-HTCC hydrogels (2 mg each) were placed on watch glasses, and 200 µL of human blood was added. For each sample, seven watch glasses were prepared. To initiate aggregation, 25 µL of 0.2 mol/L calcium chloride solution was added, and the time of clot formation onset was recorded. Every 5 min, one of the glasses was immersed in 100 mL of distilled water, and the absorbance of the resulting solution was measured at 545 nm (Q-bands of hemoglobin) using an SF-2000 spectrophotometer (LOMO Systems, St. Petersburg, Russia).

### 2.12. Statistical Assay

Each experiment was repeated at least six times. Statistical analysis was performed using the Stadia 8.0 Professional software package (http://protein.bio.msu.ru/~akula/Podr2~1.htm, accessed on 24 September 2026). Because all measured parameters followed a normal distribution, differences between control and experimental groups were assessed using Student’s *t*-test, with a significance threshold of *p* < 0.05.

## 3. Results and Discussion

### 3.1. Synthesis of Cross-HTCC Hydrogels

Cross-HTCC hydrogels were prepared in two stages. The first stage involved the quaternization of chitosan, after which the resulting products were chemically crosslinked using 1,4-butanediol diglycidyl ether.

*N*-(2-Hydroxypropyl)-3-trimethylammonium chitosan was synthesized under heterogeneous conditions in an aqueous medium by modifying chitosan with glycidyltrimethylammonium chloride. This approach belongs to polymer-analogous transformations of chitosan, so the molecular weight of the polysaccharide is assumed to remain unchanged. The resulting samples differed visually in color.

Product yields ranged from 37.6% to 79.0% (Table 1). Notably, the yield of HTCC decreased as the amount of GTMAC added increased. This is attributed to a deficit in the number of amino groups available for substitution relative to the amount of GTMAC used. As a result, most of the GTMAC cannot react with chitosan and is removed from the target HTCC during purification.

**Figure 1:**
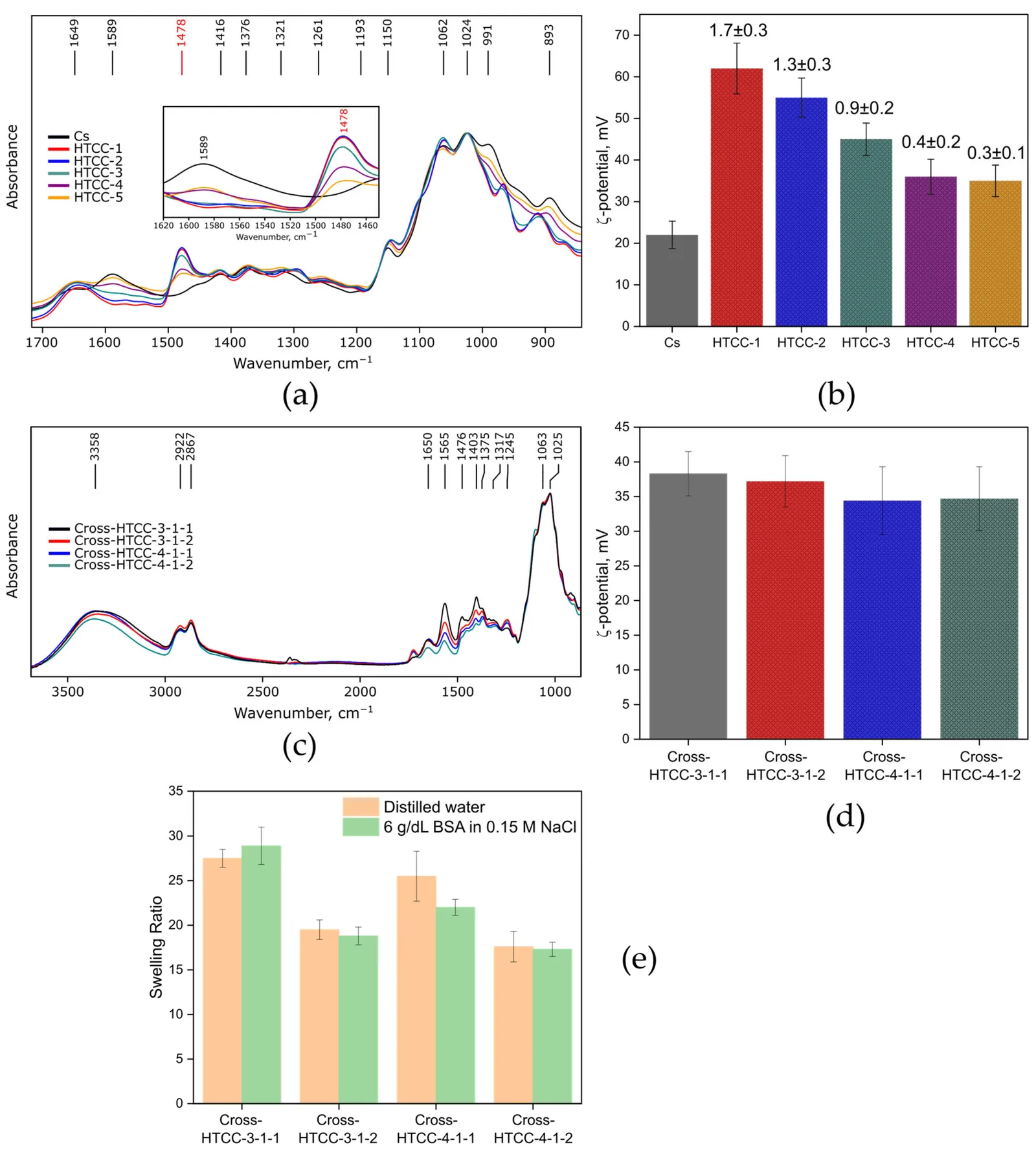
Characterization of HTCCs and Cross-HTCC hydrogels: FTIR spectra ((**a**) red font is for new FTIR bands) and ζ-potential values (**b**) (degrees of quaternization are presented above the bars) of HTCCs; FTIR spectra (**c**) and ζ-potential values (**d**) of Cross-HTCC hydrogels; swelling ratios of Cross-HTCC hydrogels (**e**).

FTIR spectroscopy was used to confirm the structure of the resulting HTCCs. Figure 1a shows the FTIR spectra of pristine chitosan and HTCC in the 850–1700 cm^−1^ range. The FTIR spectrum of pristine chitosan contains several bands in the 1700–1500 cm^−1^ region, which arise from overlapping vibrations of C–N, N–H, and C=O bonds of acetyl residues and primary amino groups. The band at 1649 cm^−1^ corresponds to C=O stretching of amide I. The amide II band (N–H bending), usually present at ~1550 cm^−1^, is masked in this case by the strong, closely located N–H bending band of primary amines at 1589 cm^−1^. Bending vibrations of CH_2_ and CH_3_ groups appear at 1416 and 1376 cm^−1^, respectively. The bands at 1321 and 1261 cm^−1^ correspond to C–N stretching. Narrow absorption bands at 1150 and 893 cm^−1^ are assigned to vibrations of glycosidic bonds. The broad band with a peak at 1024 cm^−1^ corresponds to skeletal bending vibrations of the pyranose rings, while the components at 1062 and 1024 cm^−1^ are attributed to C–O stretching in the pyranose ring structure [34].

The FTIR spectra of the synthesized HTCCs show a new, well-defined absorption band at 1478 cm^−1^, corresponding to bending and scissoring vibrations of the methyl groups of GTMAC [35]. The intensity of this band increases progressively from HTCC-5 to HTCC-1, consistent with the amount of GTMAC used in the synthesis. At the same time, the intensity of the absorption band at 1589 cm^−1^, assigned to N–H bending of primary amines, decreases progressively from pristine chitosan to HTCC-1 (Figure 1a, inset). This suggests a reduction in the number of free NH_2_ groups and, consequently, an increase in the degree of quaternization. Substitution occurs predominantly at the amino group, since it is a stronger nucleophile than the hydroxyl group; the reaction may also proceed at the OH group with the least steric hindrance.

^1^H NMR spectroscopy was used for the quantitative characterization of the degree of quaternization. By analyzing the signals of the protons of the GTMAC (~3.2 ppm) and polysaccharide (~2.1 and ~3.3–4.0 ppm) units, the degree of quaternization, expressed as the number of quaternary ammonium groups per monomer unit, was determined for each sample (Table 1, Figure S1). The obtained values ranged from 0.3 ± 0.1 for HTCC-5 to 1.7 ± 0.3 for HTCC-1. Notably, the degrees of quaternization of HTCC-4 and HTCC-5 were not statistically different. The ζ-potential of the HTCCs correlated with the degree of quaternization and increased steadily from 35 ± 3.9 mV for HTCC-5 to 62 ± 6.1 mV for HTCC-1 (Table 1, Figure 1b). Again, no statistically significant difference in ζ-potential was observed between HTCC-4 and HTCC-5. Therefore, HTCC-1–HTCC-4 were selected for the hydrogel synthesis.

Attempts to obtain crosslinked structures from HTCC-1 and HTCC-2 were unsuccessful. This is apparently due to their high degree of quaternization. Covalent crosslinking with 1,4-butanediol diglycidyl ether proceeds by the same mechanism as the reaction with GTMAC: the opening epoxide ring attacks the more reactive nucleophilic center, i.e., the amino group of chitosan or the sterically accessible OH group at C-6 position. In the highly substituted HTCC-1 and HTCC-2, nearly all accessible nucleophilic centers are occupied by quaternary ammonium groups, which prevents the formation of a network structure. Hydrogels Cross-HTCC-3-1-1, Cross-HTCC-3-1-2, Cross-HTCC-4-1-1, and Cross-HTCC-4-1-2 were therefore obtained in ~90% yield. These hydrogels differ in the degree of quaternization of the pristine HTCC-3 and HTCC-4, as well as in crosslinker content: the HTCC:BDDE molar ratio was 1:1 for the 1-1 series and 1:2 for the 1-2 series.

Figure 1c shows the FTIR spectra of the synthesized Cross-HTCC hydrogels. The spectra contain bands corresponding to the functional groups of HTCC, but some changes in their position and shape are observed. For example, the amide II band at 1565 cm^−1^ is now clearly resolved. At the same time, the trend in the intensity of the band at 1476 cm^−1^, assigned to bending and scissoring vibrations of the methyl groups of GTMAC, is preserved. Overall, while the total spectral intensity is essentially the same for all samples, the intensity of the bands describing the vibrations of the chitosan amino group (1565 cm^−1^) and the GTMAC methyl groups (1476 cm^−1^) is lower for the samples prepared with a twofold excess of BDDE. This confirms the formation of the Cross-HTCC hydrogels.

The results of ζ-potential measurements of the aqueous dispersions of the Cross-HTCC hydrogels are presented in Figure 1d. The ζ-potential values decreased compared with the corresponding HTCC precursors; however, the values for the Cross-HTCC-3 hydrogels were higher than those for the Cross-HTCC-4 hydrogels. This decrease in ζ-potential is apparently due to the fact that, during hydrogel synthesis and purification, some of the ammonium groups are converted to their base form (R_4_N^+^Cl^−^ + H_2_O ⇄ R_4_N^+^OH^−^ + H^+^ + Cl^−^), which is characterized by a lower degree of dissociation. Nevertheless, the obtained values are still sufficient to maintain the stability of the dispersed particles (>30 mV) and to enable electrostatic interactions with the negatively charged enzyme.

Figure 1e shows the swelling ratios of the Cross-HTCC hydrogels in distilled water and in a BSA saline solution mimicking the blood environment. Gravimetric analysis revealed that all hydrogels swelled more extensively in the BSA saline solution, which may be attributed to protein interactions with the hydrogel surface. BSA adsorption usually increases surface hydrophilicity [36] and, via the Donnan effect, may raise the internal ionic strength, further promoting water uptake. In addition, the samples prepared with a higher BDDE content (1–2 series) exhibited lower swelling ratios, reflecting the greater rigidity of their 3D network. No statistically significant difference in swelling ratio was found between hydrogels derived from the two precursors (HTCC-3 and HTCC-4). Overall, all samples swelled substantially—up to 28.9-fold in the albumin solution and up to 27.5-fold in water—highlighting their strong potential as hemostatic agents.

In summary, a series of HTCC samples with varying degrees of quaternization was successfully synthesized, and their structure and composition were confirmed by FTIR and ^1^H NMR spectroscopy. Crosslinking with BDDE yielded four Cross-HTCC hydrogels, whose properties depended on both the degree of quaternization of the precursor and the crosslinker content. The resulting hydrogels exhibited ζ-potential values sufficient for colloidal stability and swelling ratios of up to 28.9-fold, confirming their promise as hemostatic materials.

### 3.2. Ficin Immobilization

Optimizing the enzyme immobilization conditions is critical for creating a protein–polysaccharide system that maintains high enzyme activity. We therefore examined the following parameters for ficin immobilization on Cross-HTCC hydrogels: the pH of the sorption medium, the contact time between the enzyme solution and the hydrogels, and the dependence of enzyme activity on its content in the immobilized preparation.

#### 3.2.1. Effect of pH

**Figure 2:**
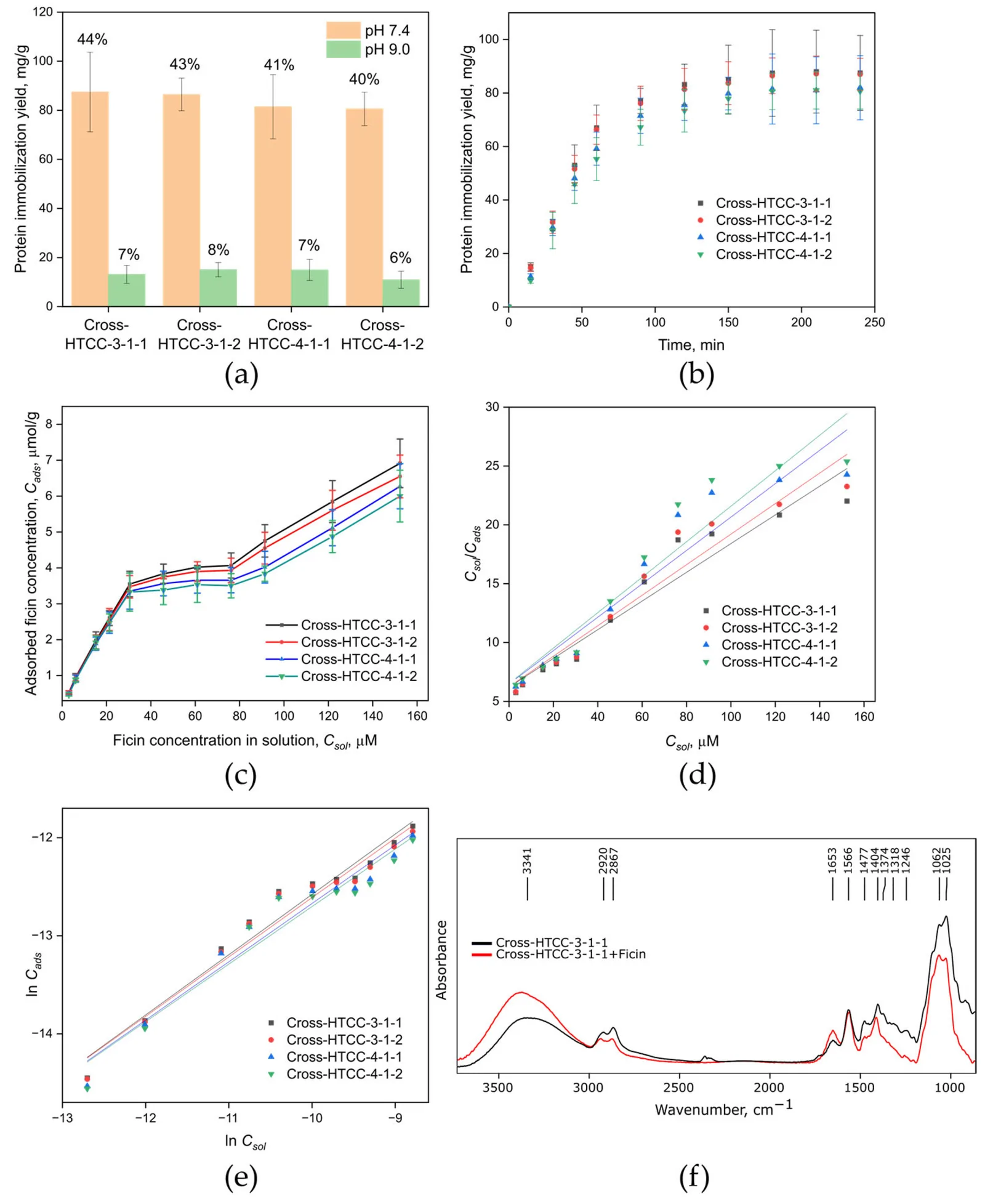
Ficin immobilization optimization: (**a**) effect of pH (*c*(ficin) = 1 mg/mL; protein immobilization yield, %, is indicated above the bars); (**b**) effect of contact time (*c*(ficin) = 1 mg/mL); (**c**) ficin adsorption isotherms at enzyme concentrations of 3–152 µM, fitted with the Langmuir (**d**) and Freundlich (**e**) models; (**f**) FTIR spectra of the Cross-HTCC-3-1-1 hydrogel and ficin immobilized on the Cross-HTCC-3-1-1 hydrogel.

Ficin is known to exhibit its highest activity in neutral and alkaline media [37]. For immobilization, we therefore selected 0.05 M Tris-HCl buffer at pH 7.4 and 0.05 M borate buffer with 0.1 M KCl at pH 9.0. The dependence of protein immobilization yield on the pH of the medium is shown in Figure 2a. At pH 9.0, which corresponds to the isoelectric point of ficin [38], the sorption efficiency did not exceed 8%, whereas at pH 7.4 it reached 44%. This indicates a substantial contribution of electrostatic interactions to the binding of ficin to the synthesized hydrogels. Notably, the type of hydrogel had almost no effect on the protein immobilization yield, which varied within 40–44% at pH 7.4 and within 6–8% at pH 9.0. This is consistent with the similar ζ-potential values of the hydrogels (Figure 1c) and further highlights the contribution of electrostatic forces to ficin adsorption onto the hydrogel surface.

**Figure 3:**
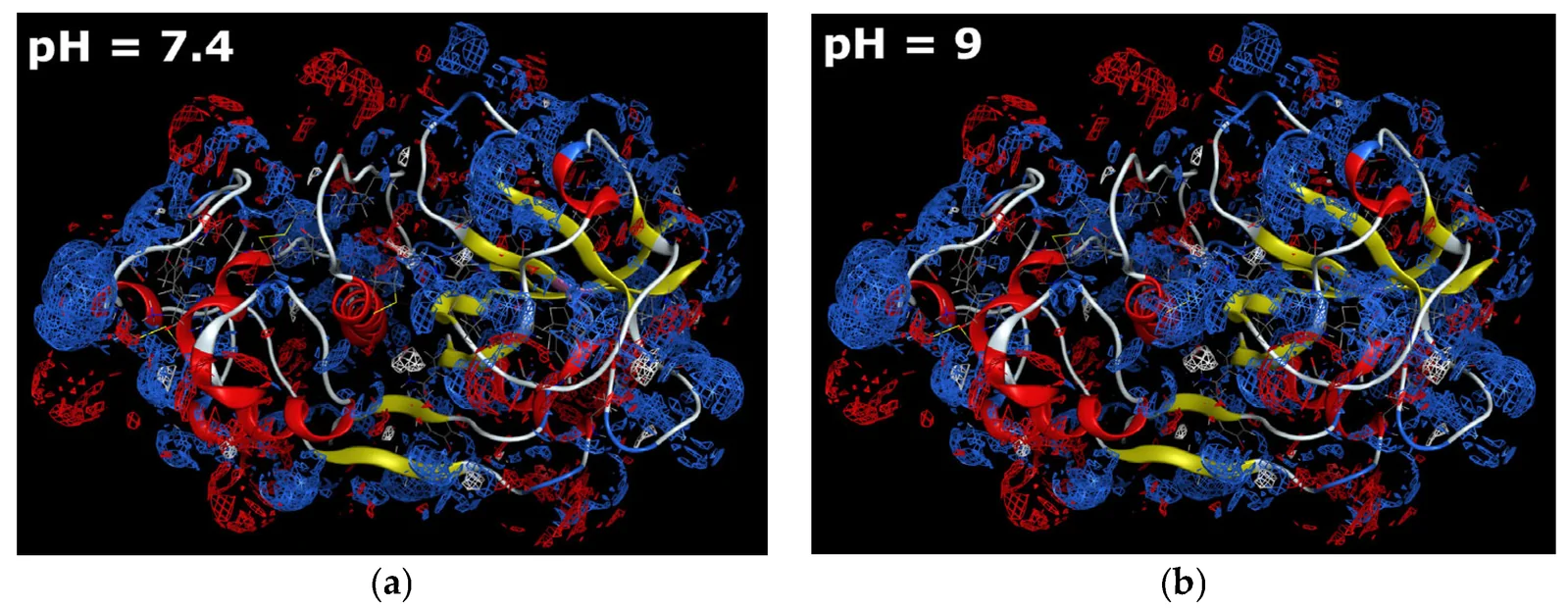
Electrostatic potential maps of ficin at pH 7.4 (**a**) and pH 9.0 (**b**). Blue and red regions indicate positively and negatively charged surface areas, respectively; white regions correspond to neutral or weakly charged areas.

To visualize how pH affects the surface charge of ficin, electrostatic potential maps of the enzyme were generated at pH 7.4 and pH 9.0 (Figure 3). At pH 7.4, which is below the isoelectric point of ficin (~9.0), the protein carries a pronounced net positive charge, reflected in the prevalence of positively charged regions (blue) on the protein surface (Figure 3a). These positively charged patches can interact electrostatically with the negatively charged groups of the hydrogel, which is consistent with the high immobilization yield observed at this pH. At pH 9.0, which is close to the isoelectric point of ficin, the net charge is substantially reduced, and the number and size of positively charged regions decrease (Figure 3b). As a result, electrostatic attraction to the hydrogel surface becomes weak, which explains the low protein immobilization yield (≤8%) observed at pH 9.0. These maps therefore support the conclusion that electrostatic interactions play a major role in ficin adsorption on the Cross-HTCC hydrogels at pH 7.4.

Based on these results, 0.05 M Tris-HCl buffer at pH 7.4 was selected for immobilization, as it ensured more efficient enzyme adsorption and because its pH matches that of human blood, which is important for the development of hemostatic agents.

#### 3.2.2. Effect of Contact Time

The kinetics of ficin interaction with the Cross-HTCC hydrogels were studied in 0.05 M Tris-HCl buffer at pH 7.4 (Figure 2b). For all Cross-HTCC hydrogels, the maximum ficin content was reached after ~180 min of contact between the enzyme solution and the hydrogels. The kinetic profiles show two distinct regions—fast sorption (0–60 min) and slow sorption (~60–170 min)—after which equilibrium was reached. Fitting the data with the pseudo-first-order and pseudo-second-order kinetic models (Figure S2, Table 2) showed that the pseudo-first-order kinetic model described the results most adequately. The rate-limiting step of ficin sorption on the studied hydrogels is therefore diffusion of ficin across the hydrogel–ficin solution interface.

The type of enzyme diffusion within the hydrogel phase was assessed by evaluating the *n_s_* value obtained from fitting the experimental data with the Ritger–Peppas model (Figure S2, Table 2). For all studied hydrogels, *n_s_* > 1, which corresponds to a special case of non-Fickian diffusion in which the diffusion rate of ficin considerably exceeds the relaxation rate of the polymer network. In other words, the swelling front advances rapidly into the hydrogel phase.

Thus, the optimal incubation time of the Cross-HTCC hydrogels in ficin solution, ensuring maximum ficin sorption, is ~180 min.

#### 3.2.3. Ficin Interaction with Cross-HTCC Hydrogels and the Effect of Ficin Content

Figure 2c shows the ficin sorption isotherms on Cross-HTCC hydrogels over a concentration range of 3–152 µM. According to the IUPAC classification, which is based on the Brunauer classification, these isotherms belong to type II, indicating polymolecular adsorption on non- or macroporous sorbents. Fitting the data with the Langmuir and Freundlich adsorption models (Figure 2d,e, Table 3) confirms this result: the coefficients of determination (R^2^) obtained for the linear fits were higher for all studied hydrogels when the Freundlich equation was applied. The sorption sites on the hydrogel surface are therefore heterogeneous —they differ in energy, meaning that ficin interacts with several types of functional groups within the polymers. This is consistent with the molecular dynamics results. For all studied hydrogels, the parameter *n* (Table 3) satisfies *n* > 1, which points to the physical nature of sorption [39], i.e., the interaction between sorbent and sorbate occurs through hydrogen bonding as well as weak electrostatic and hydrophobic interactions. This result is consistent with the earlier data on the efficiency of ficin sorption at different pH values.

To confirm the presence of heterogeneous sorption sites on the surface of the Cross-HTCC hydrogels, FTIR spectra of the immobilized ficin were analyzed (Figure 2f). Typical spectrum of ficin immobilized on Cross-HTCC-3-1-1 showed no new absorption bands compared with that of the pristine Cross-HTCC-3-1-1, but several changes were observed that confirm the interaction between the components. In the spectrum of the immobilized ficin, the intensity of the band at 1653 cm^−1^ (amide I) increased, clearly indicating the presence of protein in the sample. Changes were also observed in the intensity of the modes forming the complex band in the 1000–1100 cm^−1^ region, which describes the structural vibrations of the pyranose rings and C–OH bonds of the chitosan residues. Whereas for the pristine Cross-HTCC-3-1-1 the mode at 1025 cm^−1^ was more intense than that at 1062 cm^−1^, the immobilized preparation showed the opposite trend. This indicates loosening of the Cross-HTCC-3-1-1 structure and reorganization of the hydrogen-bond network: some intramolecular H-bonds within the hydrogel network are disrupted, and new hydrogen bonds are formed between Cross-HTCC-3-1-1 and ficin. Changes were also observed for the absorption bands in the 2850–2950 cm^−1^ region, assigned to C–H stretching. Upon interaction with ficin, the intensity of the band at 2867 cm^−1^ increased and its shape changed. This indicates the presence of hydrophobic contacts between ficin and Cross-HTCC-3-1-1.

**Figure 4:**
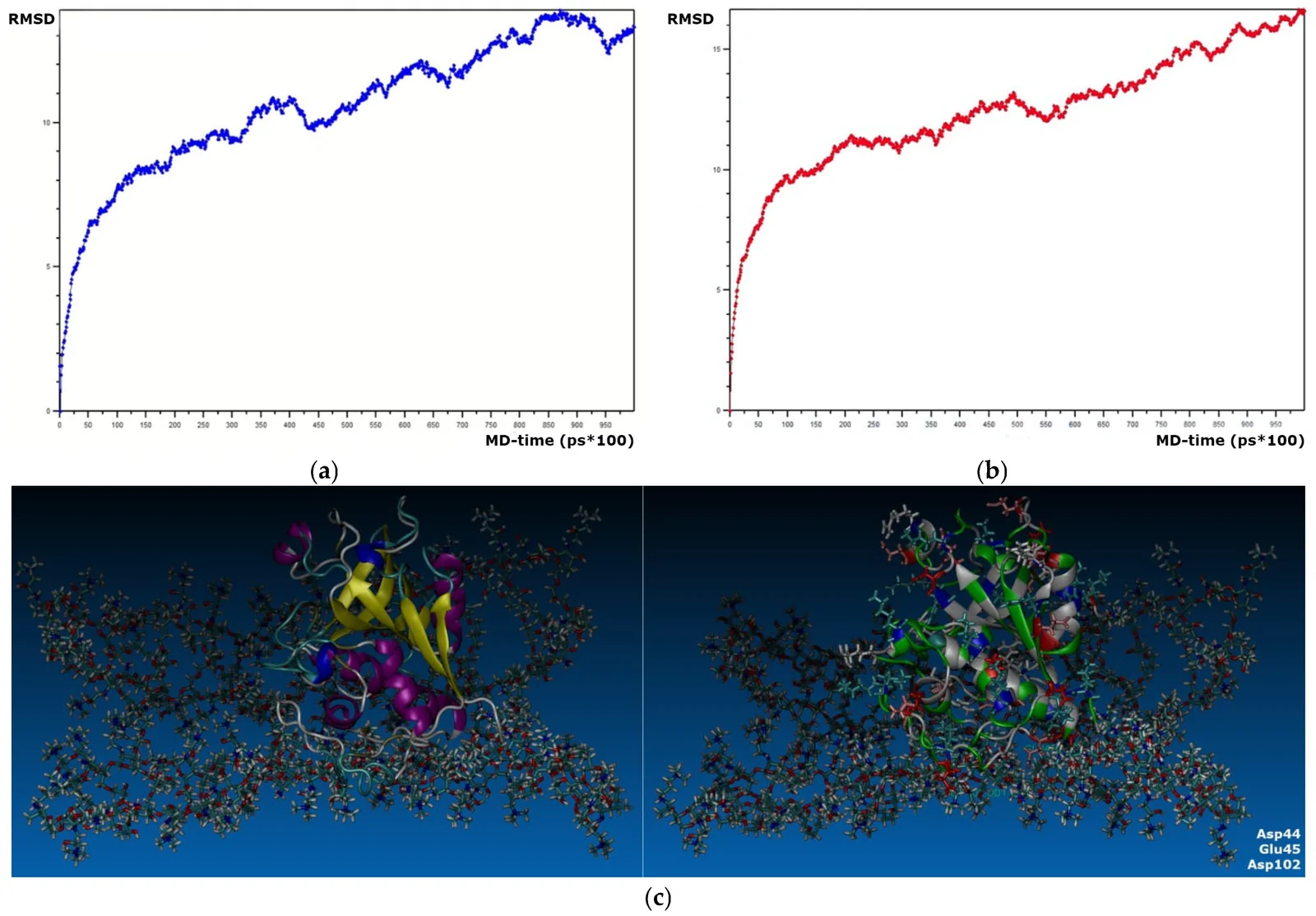
Results of MD simulation: change in RMSD values for Cross-HTCC hydrogel–water complexes: (**a**) Cross-HTCC-4-1-1 + 54,198 water molecules and (**b**) Cross-HTCC-3-1-1 + 51,238 water molecules; (**c**) structure of the modeled system, consisting of a ficin globule and Cross-HTCC-3-1-1, after 10 ns of molecular dynamics in an explicit aqueous environment. Specific amino acid residues involved in the binding of ficin to the Cross-HTCC-3-1-1 hydrogel are indicated.

The molecular dynamics simulation data are in good agreement with the FTIR data, which indicated changes in the intermolecular H-bond network of the Cross-HTCC hydrogels. The obtained MD trajectory revealed a slight loosening of the geometry of each Cross-HTCC hydrogel, as evidenced by the increase in the root-mean-square deviation (RMSD) of the hydrogel’s atoms (Figure 4a,b). Given the chemical nature of the Cross-HTCC hydrogels and the presence of polar groups in their structure, a large number of hydrogen bonds would be expected in the complexes they form. The MD trajectories showed that this number is high and fluctuates only slightly throughout the 10 ns simulation.

The 10 ns MD trajectories further revealed that the ficin globule undergoes some strengthening and compaction in the absence of ions and at pH 7.0 (i.e., under conditions close to optimal for this enzyme), whereas the system undergoes slight loosening upon the addition of the Cross-HTCC hydrogels. This compaction appears to be driven by hydrogen bonds involving the side groups of charged amino acid residues in the peripheral layer of the globule. In addition to promoting compaction, these bonds participate in alternative hydrogen bonding that ensures the high thermal stability of ficin. The theoretical principles underlying this process are well documented in the literature [40]. Notably, upon binding to the Cross-HTCC hydrogels, the enzyme’s active site remains exposed to the solution and accessible to the substrate.

A detailed study of these interactions made it possible to identify the specific amino acid residues responsible for binding to the Cross-HTCC hydrogels: in the ficin globule, these are Lys39, Asp43, Glu44, and Glu172 (Figure 4c).

**Figure 5:**
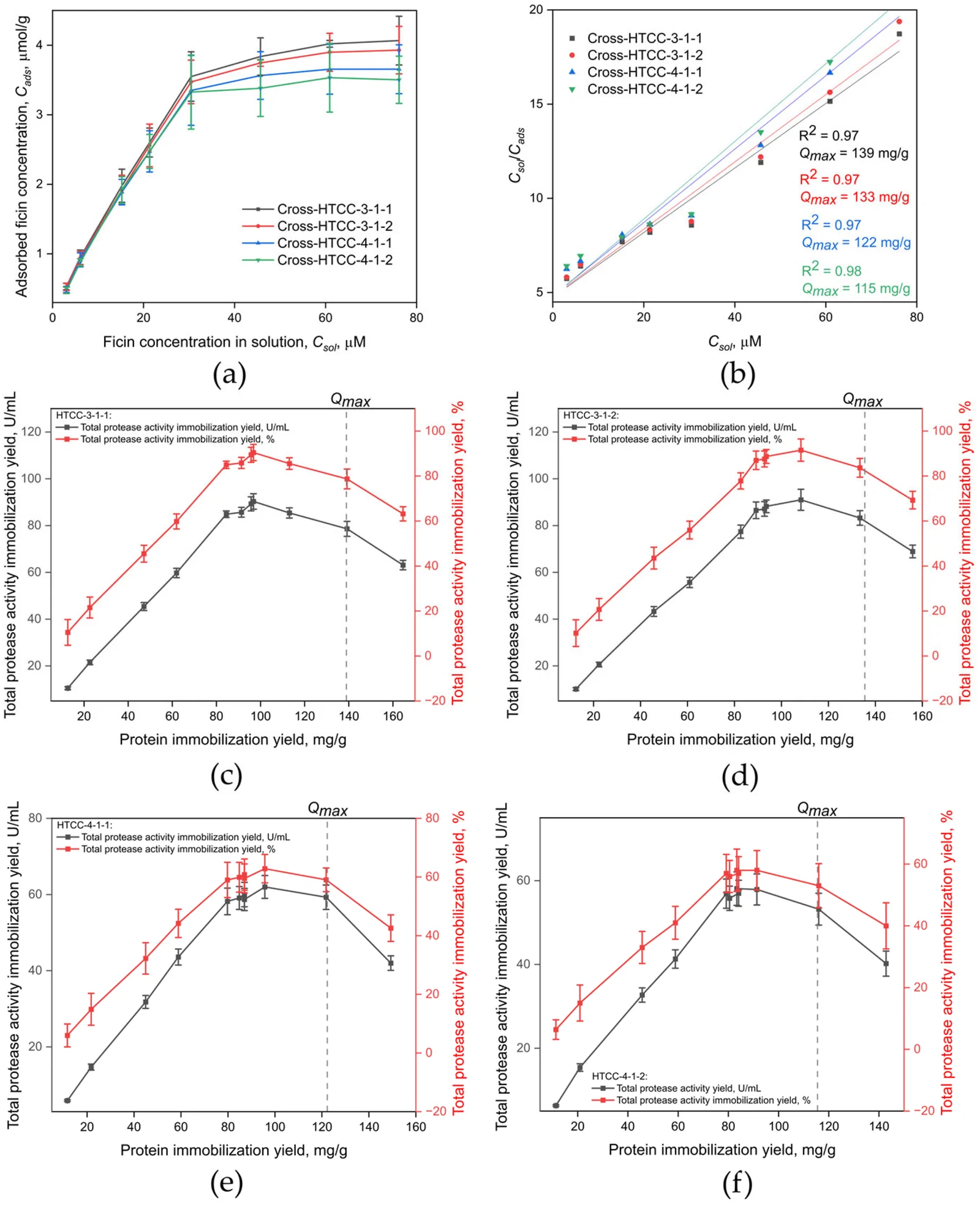
Ficin immobilization optimization: (**a**) ficin adsorption isotherms at enzyme concentrations of 3–80 µM and (**b**) their fitting with the Langmuir model; dependence of total protease activity yield on protein immobilization yield for ficin immobilized on Cross-HTCC-3-1-1 (**c**), Cross-HTCC-3-1-2 (**d**), Cross-HTCC-4-1-1 (**e**), and Cross-HTCC-4-1-2 (**f**).

Proteases are well known to undergo autolysis. To preserve enzyme activity, it is therefore advisable to prepare enzyme formulations with a protein content that minimizes contact between enzyme macromolecules. To this end, the ficin isotherms obtained over the concentration range of 3–80 µM were fitted with the Langmuir equation to determine the monolayer capacity of ficin formed on the surface of each Cross-HTCC hydrogel (*Q_max_*; Figure 5a,b). In this low-concentration range, monolayer adsorption dominates, which makes the Langmuir model appropriate for estimating the saturated monolayer loading. The experimental data were well described by this model (R^2^ > 0.97), yielding an optimal enzyme content of no more than 115–139 mg/g depending on the hydrogel type. Within these protein concentration ranges, immobilized ficin is expected to show maximum activity because autolysis is minimized.

The dependence of total protease activity immobilization yield on protein immobilization yield (Figure 5c–f) shows that, for all studied systems, the activity of the immobilized enzyme increases in the range of ~10–80 mg of protein per gram of hydrogel, then reaches a plateau (~80–110 mg/g), after which it declines. This decrease is most pronounced in the region where the protein content exceeds the *Q_max_* value for the corresponding hydrogel. Thus, optimizing the protein content in the immobilized enzymes by fitting the adsorption isotherms with the Langmuir model allows the amount of enzyme required to obtain a preparation with maximum activity to be predicted quite effectively.

In the subsequent experiments, immobilized ficin preparations with the maximum total protease activity immobilization yield were used (Figure 5c–f).

In summary, the pH of the sorption medium and the contact time were shown to be key parameters controlling ficin immobilization on Cross-HTCC hydrogels, with pH 7.4 and ~180 min ensuring maximum sorption. The adsorption isotherms, FTIR analysis, and molecular dynamics simulations consistently indicate that ficin interacts with heterogeneous sorption sites through hydrogen bonding, weak electrostatic, and hydrophobic interactions. Optimizing the protein content relative to the monolayer capacity *Q_max_* allows the preparation of immobilized ficin with maximum proteolytic activity while minimizing autolysis.

### 3.3. Ficin Release and Kinetic Whole Blood Clotting

For ficin to activate human coagulation factor X, it must first be released from the immobilized preparation. Understanding the release behavior of the active component is therefore essential when designing hemostatic agents, as it determines whether the enzyme can reach the wound site and participate in the coagulation cascade. Equally important is the ability of the material to promote blood clotting, since hemostatic performance depends on the interplay between the carrier, the released enzyme, and the blood components. These considerations motivated the next stage of our work, in which we studied the release of ficin from the Cross-HTCC hydrogels.

**Figure 6:**
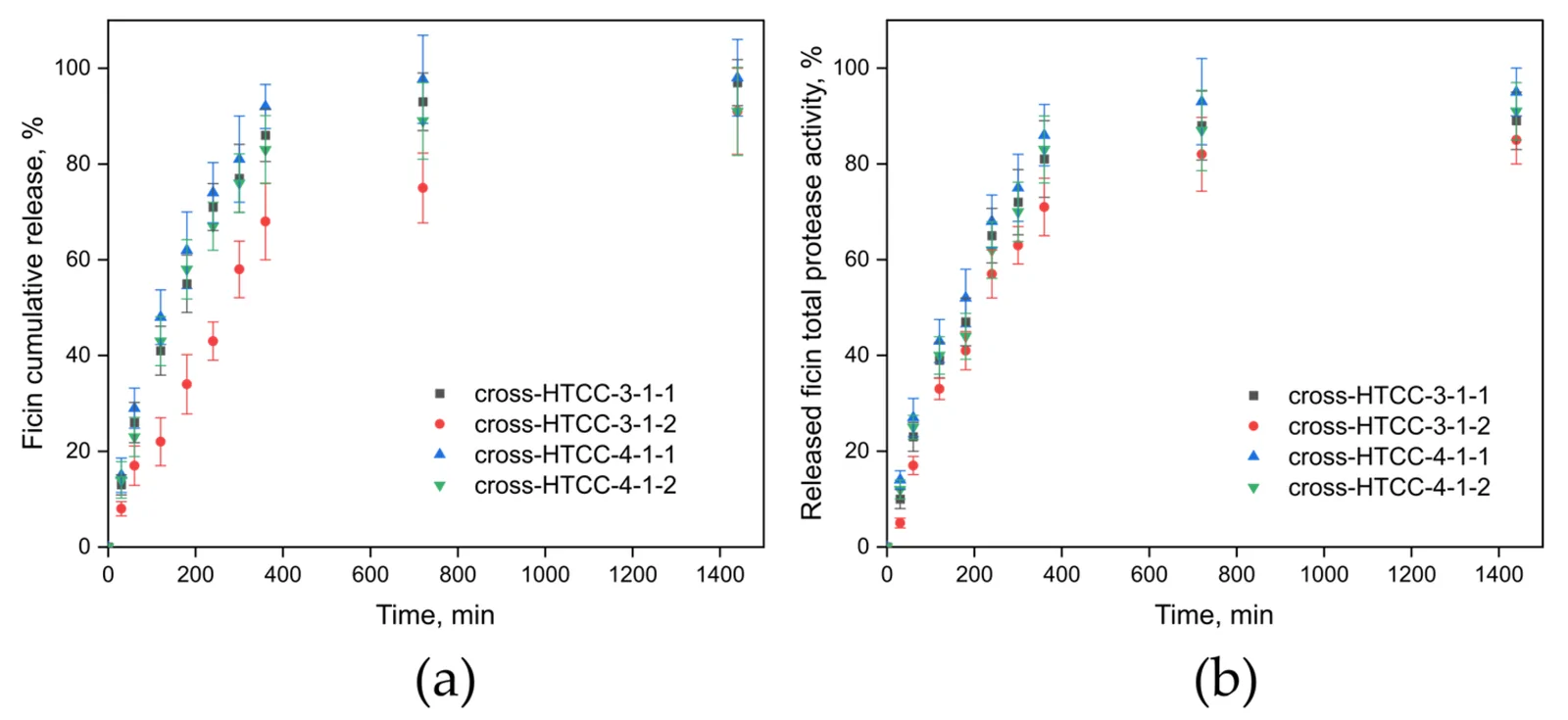
Ficin release profiles (**a**) and total protease activity of released ficin (**b**) in 0.05 M Tris-HCl buffer at pH 7.4 and 37 °C. Free ficin activity under the experimental conditions was set as 100%.

The ficin release profiles in 0.05 M Tris-HCl buffer at pH 7.4 and 37 °C are shown in Figure 6a. The obtained compositions were able to release up to 97% of the sorbed enzyme within 24 h, which corresponds to the maximum application time of a hemostatic agent. Furthermore, in all studied Cross-HTCC hydrogels, the proteolytic activity of the release medium increased with the amount of ficin released (Figure 6b). The release process was adequately described by formal first-order kinetics (R^2^ > 0.95), indicating that the release rate depends on the residual ficin concentration in the hydrogel and slows down as this concentration decreases (Figure 6a, Table 4).

To identify the driving force of release and the type of ficin diffusion, the experimental data were fitted with the Korsmeyer–Peppas model, in which the exponent *n_r_* indicates the type of release mechanism (Figure S3, Table 4). For all studied systems, 0.45 < *n_r_* < 0.89, indicating that release is governed by a combination of ficin diffusion and hydrogel erosion caused by partial hydrolysis of the crosslinks and by the chitosanolytic activity of the enzyme.

**Figure 7:**
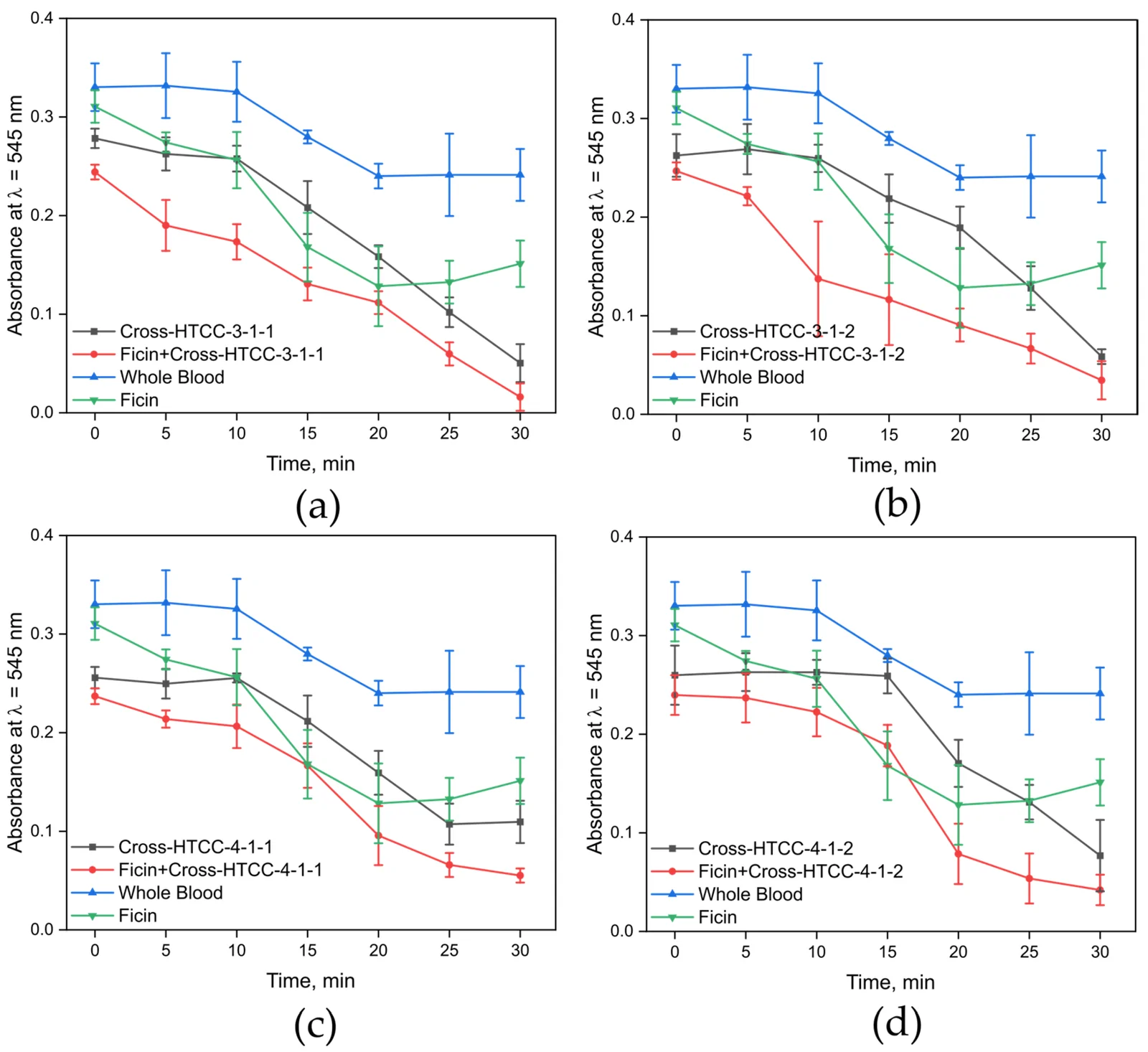
Kinetic blood clotting by optimized systems based on Cross-HTCC-3-1-1 (**a**), Cross-HTCC-3-1-2 (**b**), Cross-HTCC-4-1-1 (**c**), and Cross-HTCC-4-1-2 (**d**) hydrogels.

Figure 7 shows the kinetic blood coagulation profiles for the Cross-HTCC hydrogels and their ficin-containing counterparts. The absorbance at 545 nm was monitored over 30 min as a measure of blood cell aggregation: as coagulation proceeds, the absorbance decreases. For all studied systems, the absorbance declined steadily with time, reflecting the progressive formation of blood clots. Notably, the ficin-containing compositions produced a much steeper initial drop in absorbance than the corresponding ficin-free hydrogels, indicating faster blood cell aggregation and accelerated clot formation. This effect was consistent across all four hydrogel types, although the magnitude of the decrease varied between samples. Among the tested compositions, Ficin + Cross-HTCC-3-1-1 and Ficin + Cross-HTCC-4-1-1 showed the most pronounced reduction in absorbance, reaching the lowest values by the end of the experiment. The difference between the ficin-free and ficin-containing hydrogels became evident within the first 5–10 min, which points to the rapid involvement of the immobilized enzyme in the coagulation process. Kinetic turbidimetric assays of this type are widely used to evaluate the procoagulant performance of hemostatic materials, as the decrease in absorbance reflects the aggregation and sedimentation of blood cells during clot formation [41]. Overall, the results confirm that the incorporation of ficin into the Cross-HTCC hydrogels substantially enhances their hemostatic performance, likely due to the ability of the released enzyme to activate the natural coagulation cascade. It should be emphasized, however, that this work is limited to in vitro whole-blood phenotypic observations, and that direct factor X activation is a literature-reported intrinsic property of ficin [20] rather than a mechanism directly demonstrated here.

The controls allow the contribution of the immobilized enzyme to be separated from the intrinsic clotting behavior of blood and from the effect of free ficin. Whole blood without any added material showed only a slight and limited decrease in absorbance, reaching a T50% (50% decrease in absorbance) value beyond the 30-min observation window. This confirms that the spontaneous coagulation of donor blood was relatively slow under the assay conditions. Free ficin produced a steeper initial drop than whole blood, with an onset time of approximately 6.6 min and a T50% of approximately 17.7 min, indicating that the enzyme itself accelerates coagulation. However, the ficin-loaded hydrogels consistently outperformed free ficin, with onset times of 2.5–5.2 min and T50% values of 12.0–14.5 min. This comparison suggests that immobilization not only preserves the procoagulant activity of ficin but may also enhance its effect, possibly by concentrating the enzyme at the hydrogel–blood interface and providing a sustained release profile.

To quantify the observed differences in coagulation kinetics, two parameters were extracted from the absorbance–time curves by linear interpolation: the onset time (10% decrease in absorbance) and T50%. The corresponding values are summarized in Table 5. Quantitative analysis of the kinetic coagulation curves revealed that the ficin-containing hydrogels accelerated clot formation compared with the pristine hydrogels and free ficin. The onset time was 2.5–5.2 min for the ficin-loaded compositions, compared with 6.8–7.6 min for the pristine hydrogels and 6.6 min for free ficin. The time to 50% reduction in absorbance (T50%) was 12.0–14.5 min for the ficin-containing hydrogels, compared with 23.0–24.0 min for the pristine hydrogels and 17.7 min for free ficin. Whole blood showed the slowest coagulation, with an onset time of 21.8 min and a T50% exceeding 30 min. This nearly twofold reduction in T50% relative to the pristine hydrogels, and a 1.2–1.5-fold reduction relative to free ficin, quantitatively confirms the enhanced hemostatic performance of the ficin-loaded hydrogels.

In summary, the Cross-HTCC hydrogels released up to 97% of the sorbed ficin within 24 h, following formal first-order kinetics, while the Korsmeyer–Peppas exponent indicated a combined diffusion- and erosion-controlled mechanism. The released enzyme markedly accelerated blood clotting compared with the ficin-free hydrogels, confirming that ficin retains its activity after immobilization and is capable of activating the natural coagulation cascade. These results highlight the potential of ficin-loaded Cross-HTCC hydrogels as multifunctional hemostatic materials.

## 4. Conclusions

In this study, a series of HTCC samples with varying degrees of quaternization was synthesized and used to prepare Cross-HTCC hydrogels with different crosslinker contents. The structure and composition of the obtained materials were confirmed by FTIR and ^1^H NMR spectroscopy, and their ζ-potential and swelling behavior were characterized.

Ficin was successfully immobilized on the Cross-HTCC hydrogels by adsorption. The immobilization efficiency was strongly pH-dependent, reaching 44% at pH 7.4 versus only 8% at pH 9.0, which points to the dominant contribution of electrostatic interactions. Maximum ficin sorption was achieved after ~180 min, and the process was best described by pseudo-first-order kinetics, indicating diffusion across the hydrogel–solution interface as the rate-limiting step.

The adsorption isotherms, FTIR analysis, and molecular dynamics simulations consistently showed that ficin interacts with heterogeneous sorption sites through hydrogen bonding, weak electrostatic, and hydrophobic interactions. Optimizing the protein content relative to the monolayer capacity *Q_max_* (115–139 mg/g depending on the hydrogel) allowed immobilized ficin preparations with maximum proteolytic activity to be obtained while minimizing autolysis.

The obtained compositions released up to 97% of the sorbed enzyme within 24 h, following formal first-order kinetics, with a combined diffusion- and erosion-controlled mechanism. Importantly, the ficin-containing hydrogels markedly accelerated blood clotting compared with their ficin-free counterparts, confirming that the immobilized enzyme retains its activity and is capable of activating the natural coagulation cascade.

Overall, the developed ficin-loaded Cross-HTCC hydrogels combine efficient enzyme loading, sustained release, and enhanced hemostatic performance. These results highlight their potential as multifunctional hemostatic materials and provide a basis for further in vivo evaluation and optimization for clinical translation.

The present study deliberately focuses on carrier synthesis, ficin loading, and hemostatic performance. A comprehensive assessment of hemocompatibility and cytotoxicity, together with a systematic comparison of linear HTCC and its crosslinked hydrogel forms, is currently in progress in our group and will be reported separately. These follow-up studies are expected to provide a complete picture of the biosafety and functional potential of quaternized chitosan-based hydrogels and to support their further development toward clinical applications.

## Figures and Tables

**Table 1:** Synthesis and characterization of HTCC with different degrees of quaternization.

Sample Code	*n* (Cs):*n* (GTMAC) Molar Ratio	*m* (GTMAC), g	Yield, %	Degree of Quaternization	ζ-Potential, mV
HTCC-1	1:3	8.38	37.6	1.7 ± 0.3	62 ± 6.1
HTCC-2	1:2	5.59	47.3	1.3 ± 0.3	55 ± 4.7
HTCC-3	1:1	2.79	65.6	0.9 ± 0.2	45 ± 3.9
HTCC-4	2:1	1.40	75.2	0.4 ± 0.2	36 ± 4.1
HTCC-5	3:1	0.92	79.0	0.3 ± 0.1	35 ± 3.9

**Table 2:** Kinetic parameters of ficin adsorption models.

Sample Code	Kinetic Model					
	Pseudo-First- Order		Pseudo-Second- Order		Ritger- Peppas	
	R^2^	*k*_1s_	R^2^	*k*_2s_	R^2^	*n_s_*
Cross-HTCC-3-1-1	0.98	0.03	0.93	0.008	0.99	1.14
Cross-HTCC-3-1-2	0.98	0.03	0.93	0.008	0.99	1.15
Cross-HTCC-4-1-1	0.98	0.03	0.86	0.008	0.99	1.33
Cross-HTCC-4-1-2	0.95	0.03	0.81	0.008	0.99	1.41

**Table 3:** Adsorption parameters derived from the Langmuir and Freundlich models for isotherms obtained at ficin concentrations of 3–152 µM.

Sample Code	Langmuir Model			Freundlich Model		
	*Q_max_*, μmol/g (mg/g)	*K_L_*, L/mol	R^2^	*K_F_*, L/mol	*n*	R^2^
Cross-HTCC-3-1-1	8.20 (195.3)	19,629	0.93	620	1.63	0.96
Cross-HTCC-3-1-2	7.71 (183.5)	20,788	0.94	699	1.65	0.96
Cross-HTCC-4-1-1	7.05 (167.8)	21,885	0.92	829	1.68	0.95
Cross-HTCC-4-1-2	6.64 (158.0)	23,108	0.91	925	1.70	0.94

**Table 4:** Kinetic parameters of ficin release models.

Sample Code	Kinetic Model			
	First-Order		Korsmeyer-Peppas	
	R^2^	*k*_1r_	R^2^	*n_r_*
Cross-HTCC-3-1-1	0.95	0.005	0.87	0.54
Cross-HTCC-3-1-2	0.98	0.004	0.93	0.65
Cross-HTCC-4-1-1	0.98	0.008	0.86	0.51
Cross-HTCC-4-1-2	0.95	0.006	0.86	0.52

**Table 5:** Quantitative parameters of kinetic whole-blood coagulation derived from the absorbance–time curves.

Sample Code	A_0Min_	A_30Min_	ΔA_100%_	ΔA_10%_	ΔA_50%_	Onset Time, Min	T50%, Min
Whole Blood	0.3303	0.2413	0.0890	0.0089	0.0445	~21.8	>30.0 *
Cross-HTCC-3-1-1	0.2783	0.0503	0.2280	0.0228	0.1140	~6.8	~23.5
Cross-HTCC-3-1-2	0.2625	0.0585	0.2040	0.0204	0.1020	~7.3	~24.0
Cross-HTCC-4-1-1	0.2558	0.1095	0.1463	0.0146	0.0732	~7.2	~23.0
Cross-HTCC-4-1-2	0.2598	0.0769	0.1830	0.0183	0.0915	~7.6	~23.0
Ficin	0.3106	0.1512	0.1595	0.0159	0.0797	~6.6	~17.7
Ficin + Cross-HTCC-3-1-1	0.2441	0.0159	0.2282	0.0228	0.1141	~5.2	~14.5
Ficin + Cross-HTCC-3-1-2	0.2467	0.0345	0.2122	0.0212	0.1061	~4.2	~14.5
Ficin + Cross-HTCC-4-1-1	0.2370	0.0551	0.1818	0.0182	0.0909	~2.5	~12.0
Ficin + Cross-HTCC-4-1-2	0.2397	0.0420	0.1977	0.0198	0.0989	~3.4	~12.0

* For the Whole Blood sample, the absorbance decrease did not reach 50% of the total change within the 30-min observation period.

## Data Availability

The original contributions presented in the study are included in the article/Supplementary Materials, and further inquiries can be directed to the corresponding author.

## Supplementary Materials

The following supporting information can be downloaded at https://www.mdpi.com/article/10.3390/polym18212679/s1, Calculation of the Degree of Quaternization of HTCC Samples; Figure S1: Fragments of the ^1^H NMR spectra of HTCC-1 (blue), HTCC-2 (red), HTCC-3 (green), HTCC-4 (plum), and HTCC-5 (yellow); Figure S2: Ficin sorption kinetics fitted with the pseudo-first-order model (a), pseudo-second-order model (b), and Ritger–Peppas model (c); Figure S3: Ficin release kinetics fitted with the first-order model (a) and the Korsmeyer–Peppas model (b).
