# Supplementary material for: Ficin-Loaded Quaternized Chitosan Hydrogels: A Functional Biobased Platform for Enhanced Hemostasis
Source: Polymers (Basel). 2026 Oct 28;18(21):2679. doi: 10.3390/polym18212679 (PMC13766148; doi:10.3390/polym18212679)
Supplement: Supplementary file 1 [file polymers-18-02679-s001.zip › polymers-4626535-supplementary.pdf]

Ficin-Loaded Quaternized Chitosan Hydrogels: A Functional Biobased Platform for Enhanced Hemostasis

Maria S. Lavlinskaya, Andrey V. Sorokin, Yulia A. Redko, Victoria A. Koroleva, Maxim S. Kondratyev, Yuriy F. Zuev, Marina G. Holyavka and Valeriy G. Artyukhov

Supplementary Materials

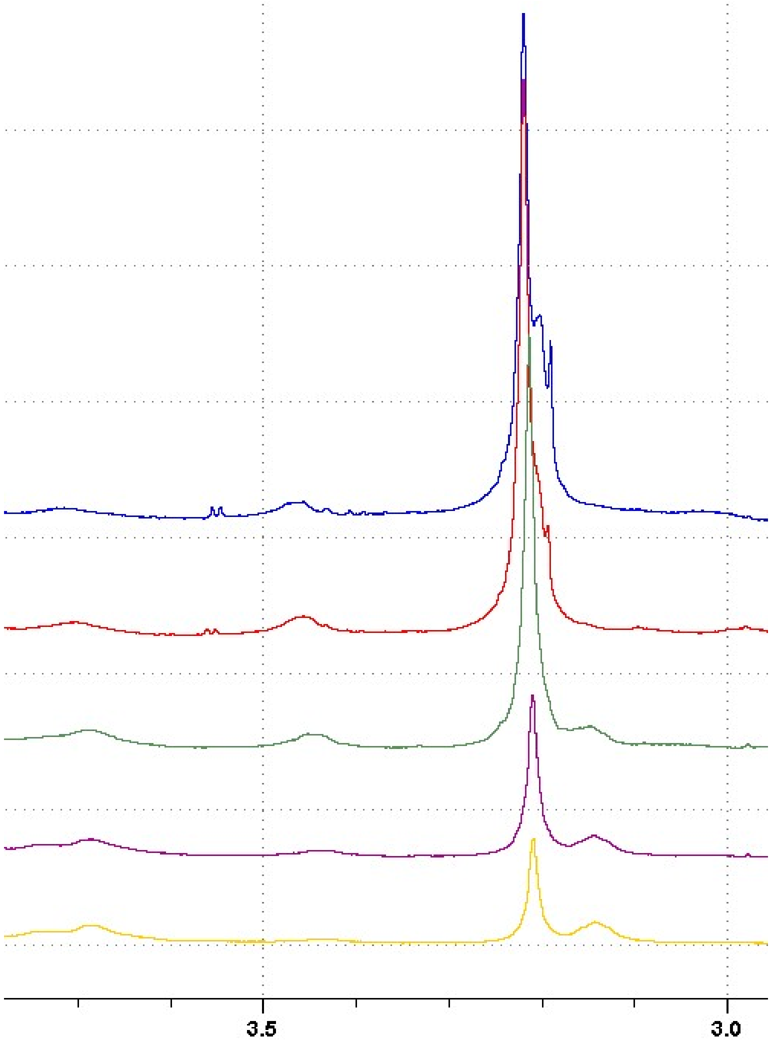

Figure S1. Fragments of the ^{1}H NMR spectra of HTCC-1 (blue), HTCC-2 (red), HTCC-3 (green), HTCC-4 (plum), and HTCC-5 (yellow).

Calculation of the Degree of Quaternization (DQ) of HTCC Samples

The DQ was calculated from the ^{1}H NMR spectra recorded with pulsed field gradient diffusion filtering, which allowed the signals of residual acetic acid to be suppressed. The integral intensities of the following signals were used:

I_1 – the signal of the methyl protons of the quaternary ammonium substituent ($N^{+}(CH_3)_3$), integrated over the range 3.0–3.3 ppm;

I_2 – the signal of the methyl protons of the *N*-acetylglucosamine units, integrated over the range 1.9–2.1 ppm;

I_3 – the signal of the main-chain chitosan protons, integrated over the range 3.3–4.0 ppm.

The DQ was then calculated as follows:

$$DQ = \frac{I_1/9}{\frac{I_3}{7 - 3 \cdot DD} + \frac{I_2}{3 \cdot DD}},$$

where $I_1/9$ corresponds to the number of quaternary ammonium groups (9 methyl protons per group), and the denominator represents the number of monomer units, taking into account the degree of deacetylation (DD) of the initial chitosan.

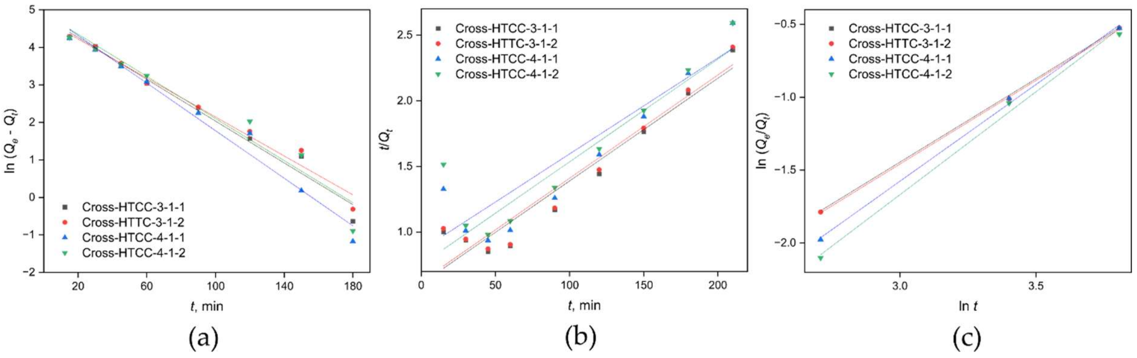

Figure S2. Ficin sorption kinetics fitted with the pseudo-first-order model **(a)**, pseudo-second-order model **(b)**, and Ritger–Peppas model **(c)**.

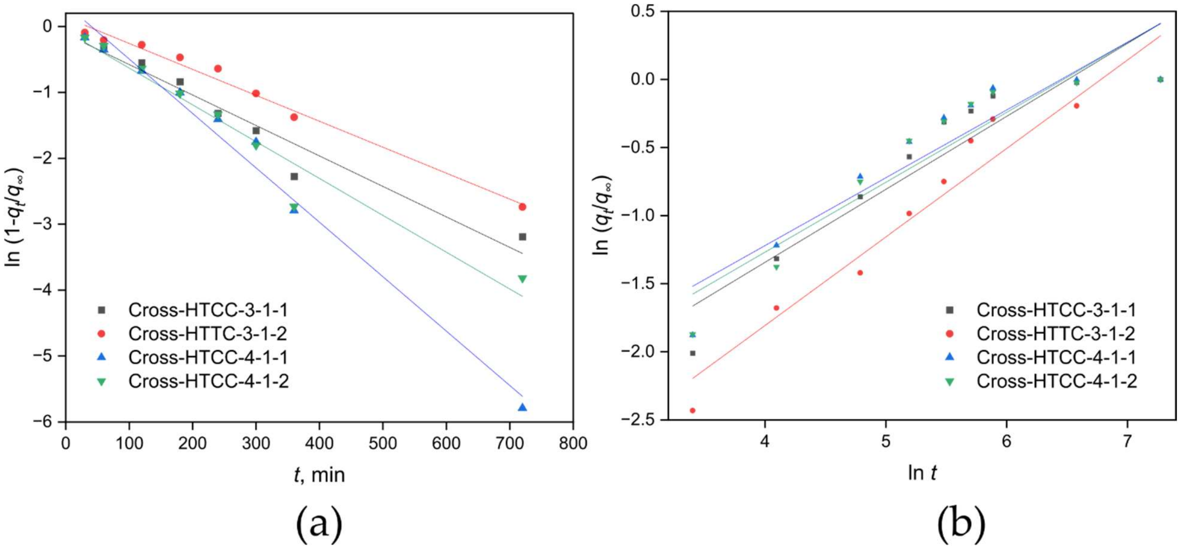

Figure S3. Ficin release kinetics fitted with the first-order model **(a)** and the Korsmeyer–Peppas model **(b)**.
